# Kaempferol regulates apoptosis and migration of neural stem cells to attenuate cerebral infarction by *O*‐GlcNAcylation of β-catenin

**DOI:** 10.1515/biol-2022-0829

**Published:** 2024-03-09

**Authors:** Song Zhang, Honglei Jiao

**Affiliations:** Department of Neurology, The Second Hospital of Hebei Medical University, No. 215, Heping West Road, Shijiazhuang, Hebei 050000, China

**Keywords:** kaempferol, Wnt/β-catenin pathway, *O*‐GlcNAcylation, MCAO, OGD/R, apoptosis

## Abstract

Ischemic stroke remains a major cause of disability and death. Kaempferol (Kae) is a neuroprotective flavonoid compound. Thus, this study aimed to explore the impact of Kae on cerebral infarction. We generated the middle cerebral artery occlusion (MCAO) mouse model to study the effects of Kae on infarction volume and neurological function. The oxygen and glucose deprivation (OGD)/reoxygenation (R) model of neural stem cells (NSCs) was established to study the effects of Kae on cell viability, migration, and apoptosis. Cell processes were assessed by cell counting kit-8, Transwell assay, flow cytometry, and TUNEL analysis. The molecular mechanism was assessed using the Western blot. The results indicated that Kae attenuated MCAO-induced cerebral infarction and neurological injury. Besides, Kae promoted cell viability and migration and inhibited apoptosis of OGD/R-treated NSCs. Moreover, OGD/R suppressed total *O*‐GlcNAcylation level and *O*‐GlcNAcylation of β-catenin, thereby suppressing the Wnt/β-catenin pathway, whereas Kae reversed the suppression. Inactivation of the Wnt/β-catenin pathway abrogated the biological functions of NSCs mediated by Kae. In conclusion, Kae suppressed cerebral infarction by facilitating NSC viability, migration, and inhibiting apoptosis. Mechanically, Kae promoted *O*‐GlcNAcylation of β-catenin to activate the Wnt/β-catenin pathway. Kae may have a lessening effect on ischemic stroke.

## Graphical abstract

**Figure:**
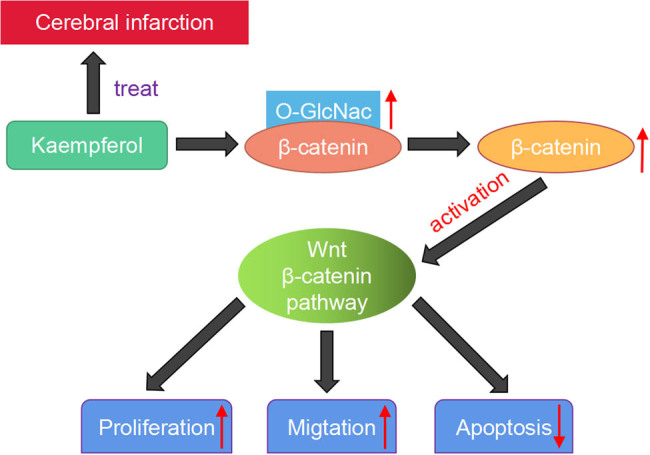

## 1 Introduction

Stroke is the fifth leading cause of death and a common cause of disability. Its prevalence in the United States is 2.5% [1]. Globally, there are more than nine million new cases each year [2]. Although its death rate has gradually declined recently, as the population ages, the lifetime risk of stroke is increasing. Cerebral infarction is the primary lesion of ischemic stroke. Ischemic stroke-induced infarction leads to neuronal damage and brain tissue death [3]. The treatment of cerebral infarction is to solve arterial occlusion and restore cerebral blood flow [4]. However, the clinical outcome is unsatisfactory. Thus, fully understanding the pathophysiological mechanism of cerebral infarction is urgent.

Kaempferol (Kae), an aglycone flavonoid, widely existed in numerous plants such as kale, spinach, chives, and tarragon, in the form of glycosides [5]. It has a variety of pharmacological activities, including anti-inflammation, anti-oxidation, anti-cancer, cardioprotective effect, and neuroprotective effect [6,7]. Based on the neuroprotective activity, Kae has been found to have beneficial effects in neurodegenerative diseases, particularly in Parkinson’s disease, Alzheimer’s disease, and ischemic stroke [8]. Previous studies have reported that Kae could alleviate mitochondrial dysfunction, inhibit neuronal apoptosis and ferroptosis, and induce autophagy [9,10,11]. However, these studies only partially explain the role of Kae, and the mechanism of Kae in cerebral infarction needs to be further elucidated.

The Wnt/β-catenin pathway plays a central role in maintaining tissue development and homeostasis [12]. The Wnt/β-catenin pathway regulates stem cell renewal, cell growth, differentiation, and metastasis [13,14]. When the Wnt protein is deficient, the β-catenin protein is maintained at low levels. Once this pathway is dysfunctional, it will cause pathological phenotypes, such as bone disease, wound healing, neurodegenerative disease, liver disease, and cancers [15,16]. The activity of the Wnt/β-catenin pathway is reduced in ischemic stroke [17]. Moreover, Kae regulates cellular processes via this signaling pathway during bone formation [18]. Thus, we speculated whether Kae participated in the pathogenesis of cerebral infarction through the Wnt/β-catenin pathway.

Based on the background, we sought to explore the effects of Kae on the Wnt/β-catenin pathway in ischemic stroke progression. We hypothesized that Kae regulated neural stem cell (NSC) injury by mediating the Wnt/β-catenin pathway. The findings will provide a novel strategy for alleviating ischemic stroke.

## 2 Materials and methods

### 2.1 Reagents

Kae (C_15_H_10_O_6_; purity ≥97%), triphenyl-2,3,5-tetrazoliumchloride (TTC), methyl 3-{[(4-methylphenyl)sulfonyl]amino}benzoate (MSAB) (Wnt/β-catenin pathway inhibitor; purity ≥95%), D-hanks, trypsin, crystal violet, paraformaldehyde, and 4,6-diamidino-2-phenylindole (DAPI) were purchased from Sigma-Aldrich (St. Louis, MO, USA). Dulbecco’s Modified Eagle Media/Ham’s F-12 (DMEM/F12), B27, epidermal growth factor (EGF), and basic fibroblast growth factor (bFGF) were purchased from Gibco (Grand Island, NY, USA). Glucose-free DMEM/F12 was purchased from Procell (Wuhan, China). Cell counting kit-8 (CCK-8) was obtained from MedChemExpress (Monmouth Junction, NJ, USA). Annexin V PE/7-AAD apoptosis detection kit, TUNEL BrightGreen apoptosis detection kit, bicinchoninic acid (BCA) protein quantification kit, and electro-chemi-luminescence (ECL) detection kit were acquired from Vazyme (Nanjing, China). Protein A/G agarose beads were obtained from Thermo Fisher Scientific (Waltham, MA, USA).

### 2.2 Animal experiment design

The animal study was approved by the Ethics Committee of The Second Hospital of Hebei Medical University (No. 2023-AE303). Male C57BL/6 mice (8–10 weeks old; Charles River, Beijing, China) were housed at 12/12 h light/dark, 20–22°C conditions with free water and food. The mice were randomly divided into three groups (five mice per group): sham, middle cerebral artery occlusion (MCAO), and MCAO + Kae groups. The mice in the MCAO group received MCAO surgery. The sham mice received the same procedure except for carotid artery occlusion. The mice in the MCAO + Kae group underwent a tail vein injection of 30 mg/kg Kae at 2 h after the MCAO procedure. The neurological function was analyzed when the mice recovered from anesthesia. All mice were sacrificed at 24 h following the MCAO process. The brain tissues were collected and washed in pre-cold normal saline to remove blood, slightly frozen in the refrigerator, and cut into five brain sections.


**Ethical approval:** The research related to animal use has been complied with all the relevant national regulations and institutional policies for the care and use of animals and has been approved by the Ethics Committee of The Second Hospital of Hebei Medical University (no. 2023-AE303).

### 2.3 MCAO model establishment

Mice were anesthetized using 1.5% isoflurane to alleviate pain. An opening was made in the middle of the mouse’s neck to expose the left common carotid artery and its branches. A silicon-coated 6–0 nylon monofilament was inserted into the common carotid artery to the opening of the middle cerebral artery to block the blood flow under a surgical microscope. A decrease in cerebral blood flow of more than 70% was observed with a Laser Doppler flowmeter (Perimed, Sweden), indicating success in MCAO. After 2 h, the monofilament was removed and the wound was closed to restore blood flow. A heating pad was used to maintain the mice’s body temperature at 37 ± 0.5°C during the whole process.

### 2.4 Neurological function analysis

The neurological status was scored using a 5-point grading method as previously described [19]: 0 point: the mouse was normal; 1 point: the contralateral front paw of the mouse could not fully extend when holding the tail; 2 points: the mouse turned around in a circle towards the ipsilateral side; 3 points: the mouse fell on the contralaterally; and 4 points: the mouse could not move autonomously.

### 2.5 TTC staining

The brain sections were incubated with 1% TTC reagent at 37°C for 0.5 h. After washing in PBS, the results were observed and photographed. The infarct region was shown in white. The total area of each brain section and its infarct size were analyzed using the Image-Pro Plus software. The infarct volume was calculated using the following formula: infarcted area/total brain area × 100%.

### 2.6 Isolation and culture of NSCs

NSCs were isolated from the cerebral cortex of fetal rats (E14). The cortex tissue was washed in D-hanks and cut into small pieces with ophthalmic scissors. The tissues were digested with 0.25% trypsin for 15 min at room temperature. After filtration with the filter (200 mesh), the cells were centrifugated at 1,000 rpm for 5 min and the supernatant was removed. The cells were maintained in DMEM/F-12 supplemented with 2% B-27, EGF (20 ng/mL), and bFGF (20 ng/mL) at 37°C with 5% CO_2_. The cells were passaged every 6–7 days. The third-passage NSCs were used in this study.

### 2.7 Oxygen and glucose deprivation (OGD)/reoxygenation (R) cell model establishment

For the OGD process, the NSCs were incubated in glucose-free DMEM/F12 under low oxygen conditions (94% N_2_, 1% O_2_, and 5% CO_2_) at 37°C for 1 h. Then, for the R process, the NSCs were incubated in the normal medium at 37°C with 95% atmosphere and 5% CO_2_ for 24 h.

### 2.8 Cell treatment

To study the role of Kae, the NSCs were exposed to 30 µM Kae for 1 h before OGD/R treatment. MSAB is a selective inhibitor of Wnt/β-catenin signaling that can bind to β-catenin to promote its degradation [20]. Therefore, to inactive the Wnt/β-catenin pathway, the NSCs were pre-treated with 10 µM MSAB for 24 h before OGD/R treatment.

### 2.9 CCK-8

A CCK-8 kit was used to analyze cell viability. After cell treatment and OGD/R induction, the CCK-8 solution was added to the plates to incubate for 4 h. The absorbance was read at 450 nm using a microplate reader (Thermo Fisher Scientific, Waltham, MA, USA).

### 2.10 Transwell assay

Cell migration was determined by Transwell assay. The 24-well chambers (8 µm pore) were purchased from Corning (Corning, NY, USA). The cell suspension was added to the top chambers, while the culture medium was added to the bottom chambers. After 24 h, the cells on the upper surface of the filter were removed, and the migrated cells were stained with crystal violet. The stained NSCs were visualized under a light microscope (Olympus, Tokyo, Japan) at 5 random fields.

### 2.11 Flow cytometry

An Annexin V PE/7-AAD kit was used to analyze cell apoptosis. The NSCs (5 × 10^5^ cells) were suspended in 1× binding buffer to make the single-cell suspension. Annexin V-PE (5 µL) and 7-AAD (5 µL) were incubated with cells for 10 min. Apoptosis was assessed using a flow cytometer (Thermo Fisher Scientific) with 1 h of adding 400 µL 1× binding buffer.

### 2.12 TUNEL assay

Cell slides were fixed with 4% paraformaldehyde and incubated with 2 µg/mL proteinase K reagent. Then, the cells were incubated with 50 µL TdT buffer (10 µL 5× Equilibration Buffer, 5 µL BrightGreen Labeling Mix, 1 µL Recombinant TdT Enzyme, and 34 µL ddH_2_O_2_) at 37°C for 1 h. After washing with PBS, the cells were re-stained with 2 μg/mL DAPI for 5 min at room temperature. The signals were visualized under a fluorescence microscope (Olympus).

### 2.13 Western blot

The NSCs were lysed using radio immunoprecipitation assay buffer on ice. Protein concentration was detected using the BCA protein quantification kit. The proteins were run using the sodium dodecyl sulfate-polyacrylamide gel electrophoresis and electrotransferred to polyvinylidene fluoride membranes. The membranes were incubated with specific primary antibodies at 4°C overnight and secondary antibodies at room temperature for 1 h. The bands were developed using the ECL detection kit.

The antibodies used were shown below: anti-*O*-GlcNAc (MA1-072, 1:1,000; Invitrogen, Carlsbad, CA, USA), anti-OGT (PA5-22071, 1:1,000; Invitrogen), anti-OGA (PA5-119277, 1:1,000; Invitrogen), anti-β-catenin (#9582, 1:1,000; Cell Signaling Technology, Danvers, MA, USA), anti-c-myc (#9402, 1:1,000; Cell Signaling Technology), and anti-cyclin D1 (#2922, 1:1,000; Cell Signaling Technology). The secondary antibodies including HRP-linked anti-mouse IgG (#7076, 1:3,000) and HRP-linked anti-rabbit IgG (#7074, 1:3,000) were purchased from Cell Signaling Technology.

### 2.14 Immunoprecipitation

The lysate of NSCs was incubated with protein A/G agarose beads at 4°C overnight. Then, the complex was incubated with anti-β-catenin or anti-IgG at 4°C overnight. After centrifugation to remove the beads, the β-catenin was detected using the Western blot.

### 2.15 Molecular docking

The 3D structure of Kae (ID: 5280863) was obtained from the PubChem database and saved in a mol2 format. The structure of human OGT (ID: 5NPS) was downloaded from the RCSB Protein Data Bank (RCSB PDB) database in the PDB format. The molecular docking was performed using the AutodockTools-1.5.6.

### 2.16 Bioinformatics

The *O*‐GlcNAcylation sites of β-catenin were predicted using the online tool (https://services.healthtech.dtu.dk/service.php?YinOYang-1.2).

### 2.17 Statistical analysis

All data in this study were analyzed using GraphPad Prism 8 software and are exhibited as mean ± standard deviation. Comparisons of difference were evaluated by Student’s *t*-test (two groups) and one-way analysis of variance (multiple groups). *P* < 0.05 was considered statistically significant.

## 3 Results

### 3.1 Kae suppresses MCAO-induced infarction and neurological injury

**Figure 1:**
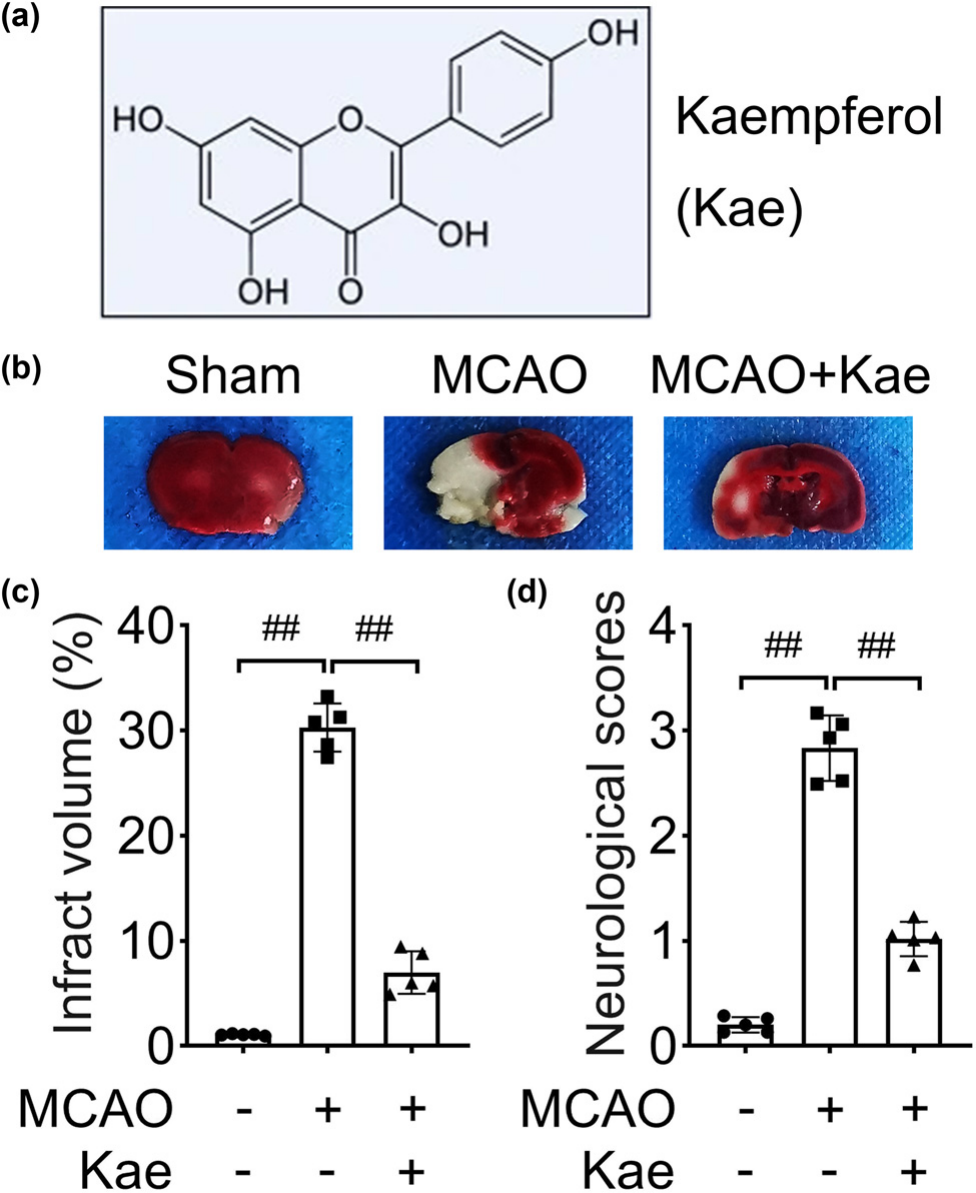
Kae suppresses MCAO-induced infarction and neurological injury. (a) The chemical structure of Kae. (b) The photograph of the brain tissues of the mice in the sham, MCAO, and MCAO + Kae group. The white is the infarct area, and the red is the normal area. (c) The infarct volume was quantified. (d) The neurological function was evaluated by neurological scores. ^##^ *P* < 0.01.

First, the MCAO mice model was established and treated with Kae to study the effects of Kae on infarction. As shown in Figure 1b and c, the infarct volume was increased in MCAO mice than in the sham mice, suggesting that the model was successfully established. Kae significantly reduced MCAO-induced infarction volume. In addition, the neurological scores of mice in the MCAO group were higher than those in the sham group, while Kae decreased the scores induced by MCAO (Figure 1d). The results indicated that Kae attenuated the cerebral infarction and neurological injury induced by MCAO.

### 3.2 OGD/R inhibits cell migration and induces apoptosis

**Figure 2:**
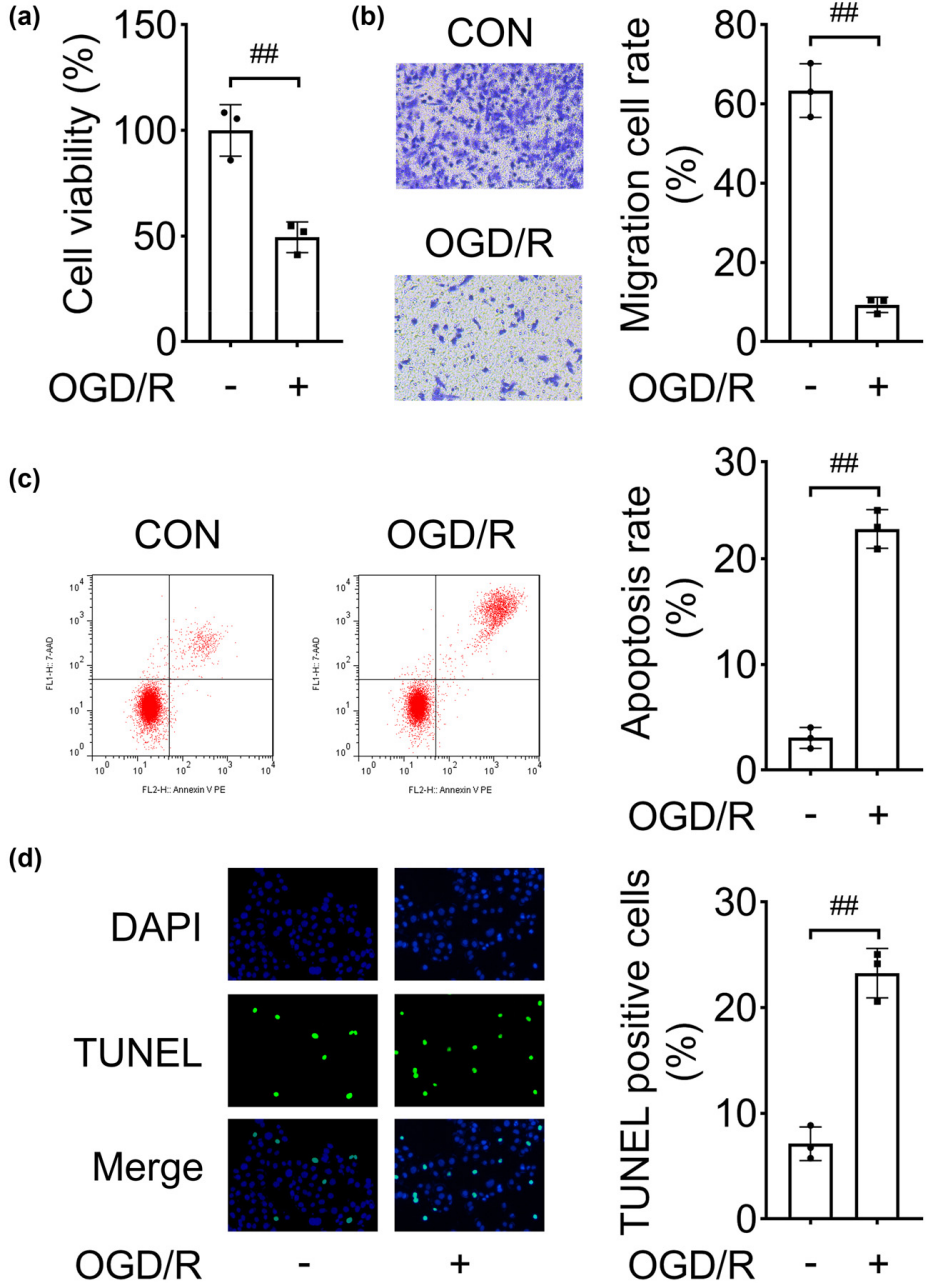
OGD/R inhibits cell migration and induces apoptosis. After the OGD/R NSCs model was established: (a) cell viability was analyzed by CCK-8; (b) cellular migration was assessed using Transwell assay; and apoptosis was evaluated by (c) flow cytometry and (d) TUNEL assay. ^##^ *P* < 0.01.

The OGD/R NSC injury model was established, and the biological functions were analyzed. Cell viability was inhibited by OGD/R treatment (Figure 2a). As compared with the control group, OGD/R suppressed cell migration (Figure 2b). Besides, according to the results of flow cytometry and TUNEL assay, ODG/R facilitated cell apoptosis, compared with the control cells (Figure 2c and d). The data suggested that the OGD/R cell model was successfully generated.

### 3.3 Kae suppresses OGD/R-induced NSC injury

**Figure 3:**
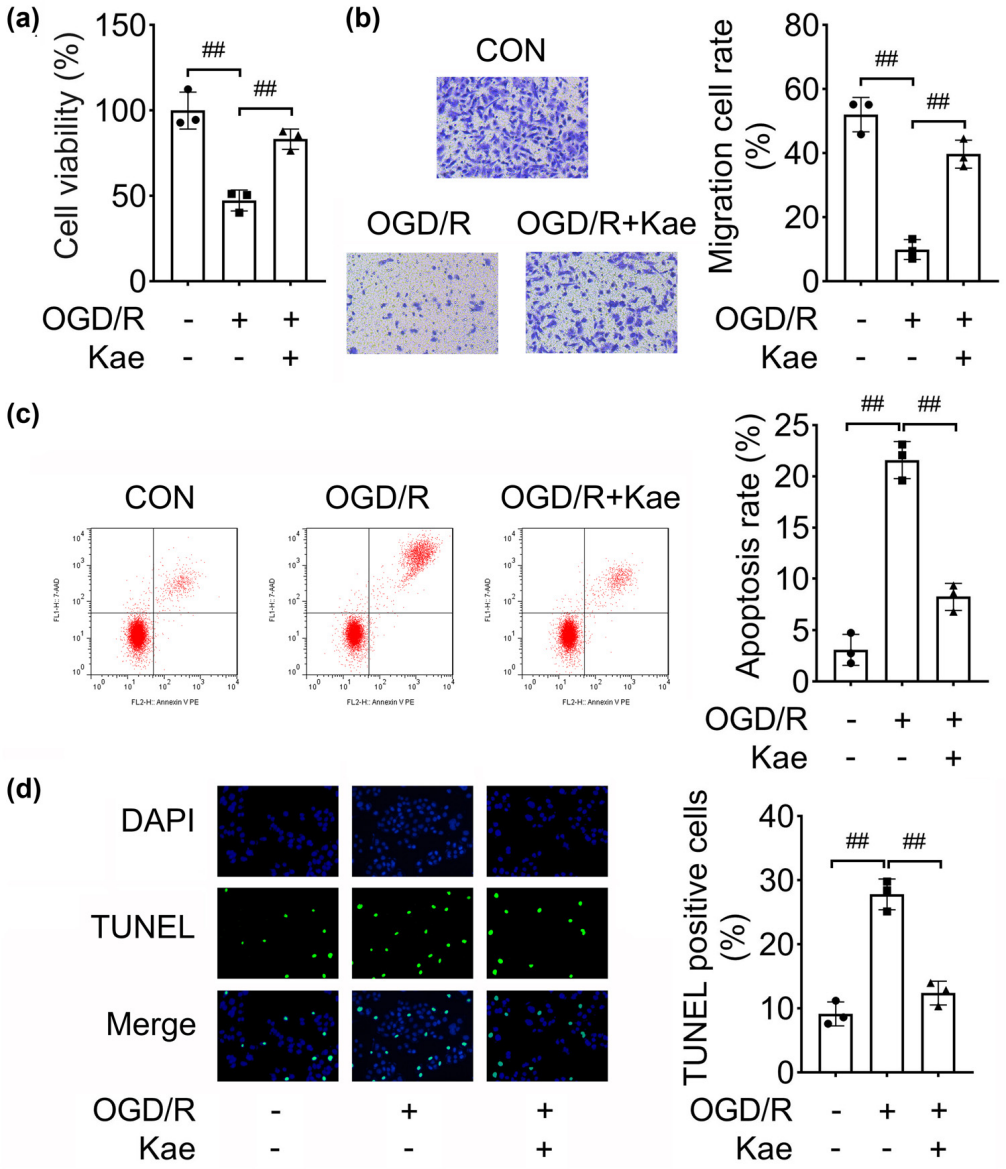
Kae suppresses OGD/R-induced NSC injury. The OGD/R cell model was established and treated with Kae: (a) CCK-8 was carried out to analyze cell viability; (b) transwell assay evaluated cell migration; and apoptosis was assessed using both (c) flow cytometry and (d) TUNEL assay. ^##^ *P* < 0.01.

To assess the function of Kae, we used Kae to treat OGD/R cells. We found that Kae promoted the cell viability of OGD/R cells (Figure 3a). Besides, the migration of OGD/R cells was facilitated by Kae treatment (Figure 3b). Inversely, cell apoptosis induced by OGD/R was abrogated by Kae (Figure 3c and d). To sum up, Kae promoted cell viability, and migration, and inhibited apoptosis of OGD/R cells, suggesting Kae attenuated NSC injury.

### 3.4 Kae promotes *O*‐GlcNAcylation of β-catenin

**Figure 4:**
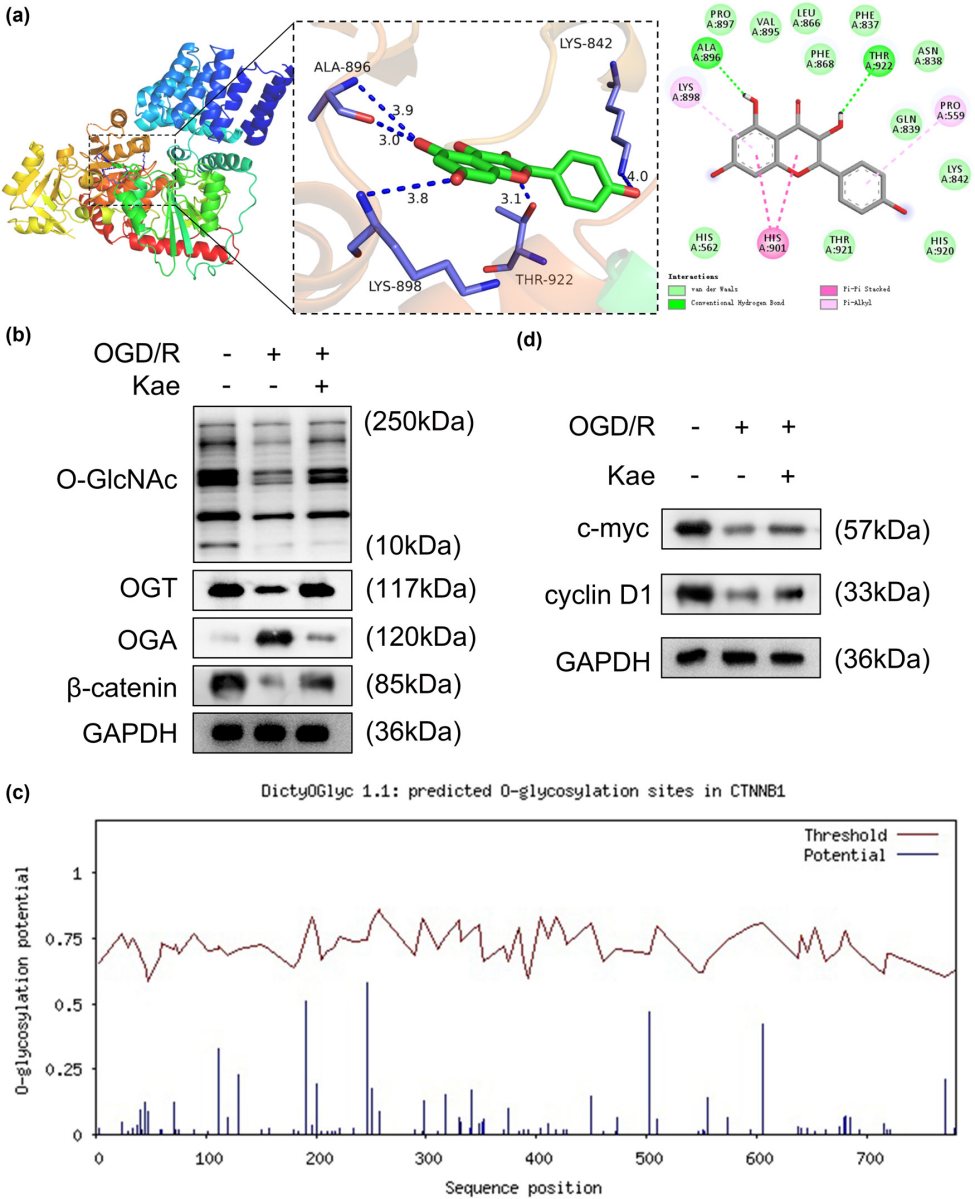
Kae promotes *O*‐GlcNAcylation of β-catenin. (a) The molecular docking between Kae and OGT. (b) The OGD/R cells exposed to Kae were lysed and the total *O*‐GlcNAc, OGT, OGA, and β-catenin levels were assessed by the Western blot. GAPDH was the endogenous control. (c) The *O*‐GlcNAcylation sites of β-catenin were predicted. (d) The protein levels of c-Myc and cyclin D1 were examined in OGD/R cells stimulated by Kae.

To explore the underlying mechanism of Kae, we performed the molecular docking between Kae and OGT. Details of the visualization of molecular docking are shown in Figure 4a. Hydrogen bond interaction force promotes the binding of molecules to active sites. The results showed that forms hydrogen bonds with OGT at ALA-896 and THR-922 sites (Figure 4a). Then, we assessed *O*‐GlcNAcylation modification. The results showed that OGD/R suppressed total *O*‐GlcNAcylation levels, whereas Kae reversed the suppression of *O*‐GlcNAcylation levels (Figure 4b). OGD/R treatment decreased the protein levels of OGT and increased OGA protein levels, as well as reduced *O*‐GlcNAcylation levels of β-catenin, while Kae reversed the OGD/R effects (Figure 4b). Moreover, multiple *O*‐GlcNAcylation sites of β-catenin were predicted (Figure 4c). In addition, the protein levels of c-Myc and cyclin D1 were downregulated by OGD/R treatment, which was abrogated by Kae treatment (Figure 4d). The data indicated that Kae promoted *O*‐GlcNAcylation of β-catenin and thus activated the Wnt/β-catenin pathway in the OGD/R NSCs.

### 3.5 Kae inhibits NSC injury by activating the Wnt/β-catenin pathway

**Figure 5:**
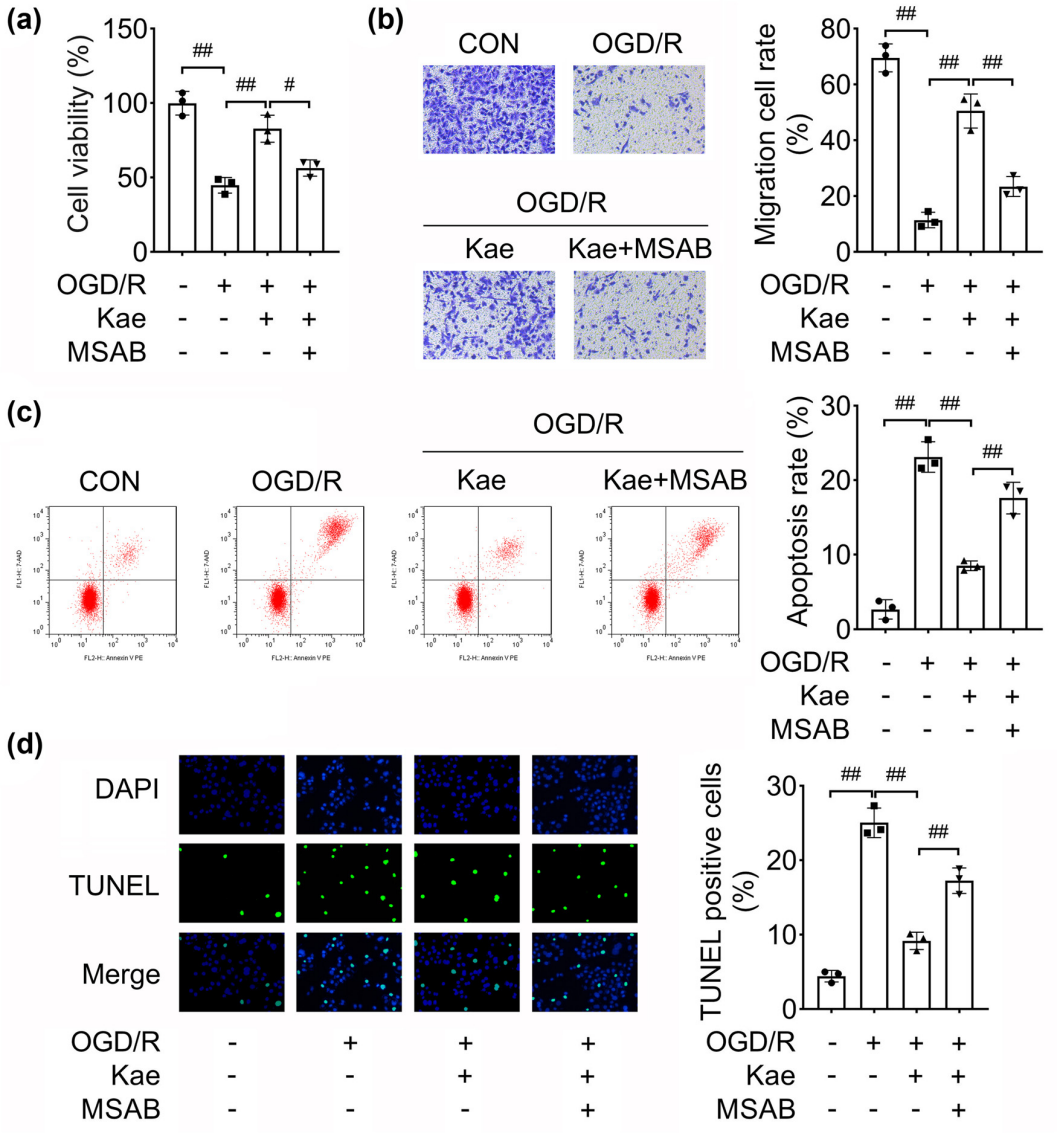
Kae inhibits NSC injury by activating the Wnt/β-catenin pathway. After the OGD/R NSCs treated with Kae and MSAB: (a) cell viability was determined using CCK-8; (b) transwell assay evaluated cellular migration; and (c) flow cytometry and (d) TUNEL assay were performed to evaluate apoptosis. ^##^ *P* < 0.01. ^#^ *P* < 0.05.

To confirm that Kae regulated the biological functions by the Wnt/β-catenin pathway, we used MSAB to suppress the activation of this pathway. MSAB counteracted the promotion of cell viability induced by Kae (Figure 5a). Kae promoted cell migration, while MSAB inhibited the cell migration of Kae-stimulated NSCs (Figure 5b). In addition, cell apoptosis suppressed by Kae was partly abrogated by MSAB (Figure 5c and d). Taken together, the inactivation of the Wnt/β-catenin pathway reversed the Kae-induced neuroprotective effect.

## 4 Discussion

Kae has various pharmacological activities, especially neuroprotective effects. Recent studies have revealed that Kae has the effect of alleviating cerebral infarction through *in vivo* study [9,10,21]. MCAO is the most widely used model to study cerebral infarction *in vivo* [22]. Herein, we used male C57BL/6 mice to establish the MCAO model. Because estrogen can induce vasodilation and has neuroprotective effects after ischemia [23,24], female mice are not suitable for modeling. The results of this study showed that Kae inhibited infarction volume and reduced the neurological score of MCAO mice, demonstrating that Kae can protect against cerebral infarction. However, the pathogenesis of cerebral infarction is complex.

NSCs have the ability to self-renewal and can differentiate into mature neurons and glia, which can change the function of the nervous system and repair the damaged nervous system [25]. Under pathological conditions, the migration of activated NSCs to adjacent injury sites is inhibited, which makes it difficult for NSCs to repair the damaged nervous system [26]. Indeed, NSCs participate in the pathogenesis of ischemic stroke. Thus, transplantation of NSCs may be a promising therapy for ischemic stroke [27]. However, our current understanding of NSCs in stroke is still very limited.

Kae has been reported to have pharmacological activity in alleviating stroke. For example, Kae inhibits inflammation, oxidative stress, and apoptosis of injuried endothelial cells [28]. Besides, Kae suppresses neuron loss and glial cell activation and inhibits neutrophil activation in peripheral blood and brain [29]. Accumulating evidence has revealed that Kae improves neuron damage by promoting cell proliferation and inhibiting LDH release of NSCs [30,31]. In this study, we focused on NSC injury mediated by Kae. We found that Kae facilitated cell viability and migration and inhibited the apoptosis of NSCs induced by OGD/R, suggesting that Kae attenuated cerebral infarction by alleviating the injury of NSCs.

*O*‐GlcNAcylation is a type of post-transcriptional modification of proteins, manifesting as the addition of the *O*-GlcNAc fragment to the serine/threonine residue of the target protein. GlcNAc transferase (OGT) and *O*-GlcNAcase (OGA) are important enzymes in the regulation of *O*‐GlcNAcylation, which adds and removes *O*-GlcNAc, respectively [32,33]. Aberrant *O*‐GlcNAcylation is involved in multiple diseases including stroke [34,35]. The promotion of *O*‐GlcNAcylation exerts neuroprotective effects and improves outcomes in ischemic stroke [36,37]. In addition, β-catenin is a developmental and homeostasis regulator that is associated with cell cycle and adhesion [38]. Meantime, it is the main effector of the Wnt signal. The activation of the Wnt pathway enhances β-catenin stability and increases the transfer into the nucleus, participating in cell biological behaviors such as proliferation, differentiation, and migration [39]. We found that Kae can form molecular docking with OGT, indicating that Kae can act on OGT. Previous studies showed that *O*‐GlcNAcylation promotes the stability of β-catenin, which is mediated by OGT and OGA [40,41]. Moreover, *O*‐GlcNAcylation of β-catenin is associated with disease progression. For example, PGM3-mediated *O*‐GlcNAcylation of β-catenin enhanced β-catenin activity, thereby facilitating colorectal cancer progression [42]. However, whether β-catenin *O*‐GlcNAcylation participated in the functions of NSCs remains unclear. In this study, the data indicated that Kae increased OGD/R-inhibited *O*‐GlcNAcylation of β-catenin, thereby activating the Wnt/β-catenin pathway. Based on the crucial role of the Wnt/β-catenin pathway in stroke, we further found that inactivation of the Wnt/β-catenin pathway reversed the promotion of cell viability and migration and the suppression of apoptosis of NSCs. Taken together, Kae attenuated the cerebral infarction by activating the Wnt/β-catenin pathway mediated by *O*‐GlcNAcylation of β-catenin.

In conclusion, Kae promoted the viability, and migration, and inhibited apoptosis of NSCs by activating the Wnt/β-catenin pathway mediated by *O*‐GlcNAcylation of β-catenin, thereby attenuating cerebral infarction and neurological injury. The findings provided a theoretical basis for Kae to be used as an effective drug for ischemic stroke therapy.
